# Comparative effects of 2 Hz vs. 100 Hz transcutaneous electrical nerve stimulation on upper limb motor function post-stroke: design and rationale for a randomized trial

**DOI:** 10.3389/fneur.2025.1641781

**Published:** 2025-09-23

**Authors:** Chengning Song, Huanxin Xie, Bo Lei, Nana Feng, Zhixian Li, Yanxian Zou

**Affiliations:** ^1^Department of Rehabilitation Medicine, Fuyong People's Hospital of Bao'an District, Shenzhen, China; ^2^Fourth Clinical Medical College, Guangzhou University of Chinese Medicine, Guangzhou, China

**Keywords:** frequency of TENS, upper limb motor function, post-stroke, protocol, randomized controlled trial

## Abstract

**Background:**

Stroke-induced upper limb dysfunction significantly impacts rehabilitation outcomes. Transcutaneous Electrical Nerve Stimulation (TENS) shows promise, but the optimal frequency for motor recovery remains unclear. This study investigates the comparative effects of low-frequency (2 Hz) and high-frequency (100 Hz) TENS in post-stroke upper limb rehabilitation. This article describes the design and conduct of this trial.

**Method/design:**

An assessor-blinded, single-center randomized controlled trial with partial participant blinding (active vs. placebo) will recruit 156 participants aged 40–80 years, 10 days to 2 months post-stroke, with mild-to-moderate upper-limb impairment (Brunnstrom III–V). Participants will be randomly assigned (1:1:1:1) to 2 Hz TENS, 100 Hz TENS, placebo TENS, or a no-TENS usual-care control for 8 weeks (3 sessions/week, 30 minutes/session). The primary outcome is the Fugl–Meyer Assessment of the Upper Extremity (FMA-UE). Secondary outcomes include Manual Muscle Testing (MMT), the Modified Ashworth Scale, electroencephalography (EEG) metrics, the Lindmark Motor Score (hand subscale), and the Barthel Index, assessed at baseline, weeks 4 and 8, and at 1 and 3 months post-intervention.

**Discussion:**

This trial is, to our knowledge, among the first randomized head-to-head comparisons of low- vs. high-frequency TENS for post-stroke upper-limb rehabilitation. Its findings are expected to clarify frequency-dependent effects, narrow the plausible range of effective parameters, and provide evidence to inform clinical guidelines and future rehabilitation strategies.

## Background

Stroke is a leading cause of long-term disability and mortality worldwide, and approximately one-third of survivors continue to experience upper limb impairment six months after the event (1–3). This persistent motor dysfunction significantly limits functional independence and quality of life, underscoring the need for effective and individualized rehabilitation strategies. Conventional interventions, including physical therapy and occupational therapy, remain the cornerstone of upper limb recovery after stroke. These approaches focus on task-specific training and motor relearning through repetitive movement practice, but their effectiveness often plateaus in the subacute phase, especially in patients with moderate to severe impairment. Pharmacological treatments, such as botulinum toxin injections, are commonly used to address spasticity, though they offer only transient benefits and carry risks such as muscle weakness and the need for repeated administration (4). In response to these limitations, emerging technologies—such as brain-computer interfaces (BCI), robotic-assisted therapy, and virtual reality—have demonstrated potential for improving motor outcomes by enhancing neuroplasticity. However, widespread clinical adoption of these techniques remains limited due to high costs, technical complexity, and accessibility issues (5).

Within this evolving therapeutic landscape, TENS has emerged as a practical, non-invasive adjunct to conventional stroke rehabilitation. Best-practice documents and reviews mainly discuss its role in modulating neuromuscular excitability and managing post-stroke spasticity, whereas evidence that it directly facilitates upper-limb motor recovery remains mixed. Despite its growing use, clinical outcomes remain variable, largely due to inconsistencies in stimulation parameters—particularly frequency (6). Both low-frequency and high-frequency TENS have been explored in clinical studies, but with conflicting results. For example, Wang et al. (7) reported no significant improvement in upper limb motor function following 2 Hz stimulation. while Sonde et al. (8) found only transient benefits with 1.7 Hz that were not sustained at long-term follow-up. In contrast, Chen et al. (9) demonstrated that 100 Hz stimulation produced significant and sustained improvements in upper limb motor outcomes. These discrepancies highlight the need for a rigorously designed trial to directly compare the frequency-specific effects of TENS in post-stroke upper limb rehabilitation.

Despite prior work, head-to-head randomized evidence directly comparing low- vs. high-frequency TENS for post-stroke upper-limb motor recovery remains limited. Addressing this gap is important for refining clinical protocols and improving patient outcomes. Accordingly, this study will systematically compare 2 Hz and 100 Hz TENS in stroke survivors to generate frequency-specific evidence to guide treatment strategies.

TENS exerts its effects through both peripheral and central mechanisms. The modality is based on the gate control theory of pain modulation (10), involving the stimulation of peripheral nerve fibers with electrical impulses. Peripherally, TENS has been shown to reduce stretch reflex excitability and prolong H-reflex latency in the affected limb (11). Centrally, it can activate motor-related cortical areas and enhance functional reorganization within the lesioned hemisphere (12). These neurophysiological effects support its therapeutic potential not only for spasticity management but also for motor restoration in stroke rehabilitation.

Clinically, TENS can be categorized by intensity and application mode: sensory-level TENS (high-frequency, low-intensity), motor-level TENS (low-frequency, high-intensity) (13), and acupuncture-like TENS, also known as transcutaneous electrical acupoint stimulation (TEAS) (14). While each modality has demonstrated benefits in various neurological disorders, the lack of consensus on optimal frequency—especially in the context of stroke—remains a key barrier to standardization. This trial seeks to provide definitive comparative evidence that may guide frequency selection and enhance the efficacy of TENS in post-stroke upper limb rehabilitation.

## Method/design

### Study design overview

**Figure 1:**
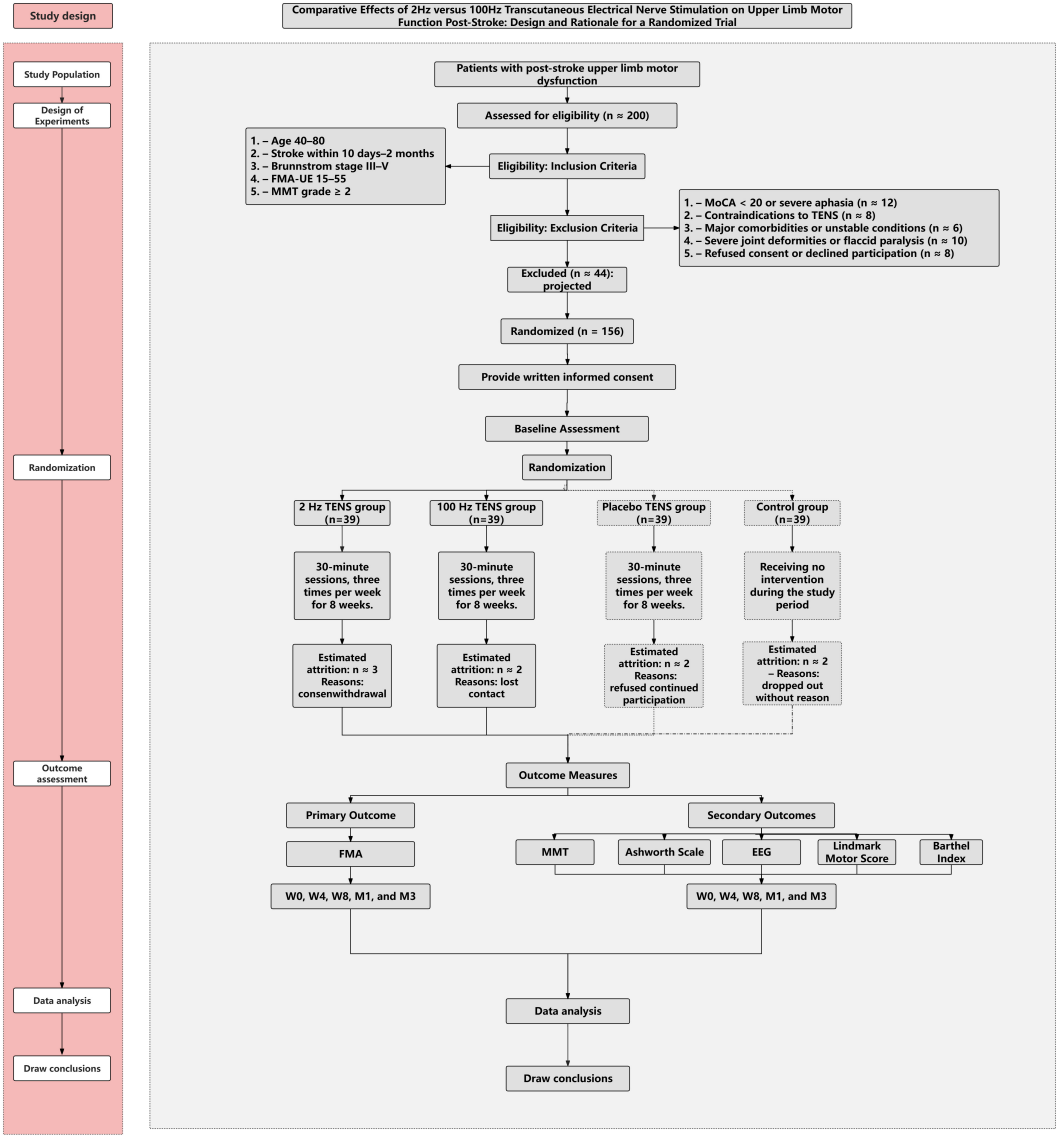
Anticipated CONSORT flow diagram for participant recruitment and allocation.

This assessor-blinded, single-center randomized controlled trial with partial participant blinding (active vs. placebo) will be conducted at the Rehabilitation Department of Fuyong People's Hospital, Bao'an District, Shenzhen, Guangdong, China. The trial will compare the effects of 2 Hz and 100 Hz TENS on post-stroke upper-limb motor dysfunction. Participants will be randomized (1:1:1:1) to 2 Hz TENS, 100 Hz TENS, placebo TENS, or a no-TENS usual-care control. The intervention will last 8 weeks, with three sessions per week (Monday, Wednesday, Friday), 30 min per session. Figure 1 depicts the study phases and anticipated participant flow.

The primary outcome is the FMA-UE, which evaluates motor control from proximal to distal segments and the transition from synergistic to isolated movements in stroke survivors (15). The FMA-UE has demonstrated excellent inter-rater (ICC = 0.997) and intra-rater reliability (ICC = 0.993) (16).

Secondary outcomes include:

1. Muscle strength of wrist flexion and extension, assessed by MMT, rated on a scale from 0 (no contraction) to 5 (normal strength). MMT has shown strong inter-rater reliability when performed by trained evaluators across various conditions (17).
2. Spasticity of the wrist flexors, measured using the Modified Ashworth Scale, which exhibits good intra-rater reliability in patients with upper limb hemiparesis (18).
3. Hand grasp function, evaluated using a subscale of the Lindmark Motor Score, a valid and reliable measure of hand motor function (19).
4. Functional independence, assessed by the Barthel Index, which evaluates performance in basic activities of daily living and is widely used in stroke research and clinical practice (20).
5. Cortical excitability and interhemispheric balance, measured using resting-state EEG. EEG signals will be recorded with standard 10–20 electrode placement, focusing on α (8–13 Hz) and β (13–30 Hz) band power at C3 and C4 electrodes. The α/β power ratio between C3 and C4 will be computed to assess hemispheric balance. These EEG markers have been widely used to reflect motor cortex activation and neuroplasticity changes following neuromodulatory interventions in stroke patients (21, 22).

Covariates include age, sex, body mass index (BMI), time since stroke onset, type of stroke (ischemic or hemorrhagic), and side of hemiplegia (left or right). Outcome assessors and the statistician will be blinded to group allocation. A detailed timeline and classification of outcome measures are provided in Table 1.

**Table 1:** Schedule of assessments and outcome measures across study timepoints.

	**Before-intervention**	**Mid-intervention**	**Post-intervention**	**1-mo follow-up**	**3-mo follow-up**
**Time (Months)**	**0**	**1**	**2**	**3**	**5**
**Baseline**					
Sex (male/female) Side of hemiplegia (left/right)	**√**				
Type of stroke (ischemia/hemorrhage)	**√**				
Age, y BMI	**√√**				
Time since stroke, y **Primary outcome variable FMA-UE(66)**	**√**				
A. Upper Extremity (36)	**√**	**√**	**√**	**√**	**√**
B. Wrist (10)	**√**	**√**	**√**	**√**	**√**
C. Hand (14)	**√**	**√**	**√**	**√**	**√**
D. Coordination/Speed (6)	**√**	**√**	**√**	**√**	**√**
**Secondary outcome variables**	**√**	**√**	**√**	**√**	**√**
**MMT**	**√**	**√**	**√**	**√**	**√**
**Ashworth Scale EEG**	**√√**	**√√**	**√√**	**√√**	**√√**
**Lindmark Motor Score**	**√**	**√**	**√**	**√**	**√**
**Barthel Index**	**√**	**√**	**√**	**√**	**√**

“√” indicates that the variable is assessed at the corresponding timepoint.

### Study sample

Based on a preliminary RCT (23) investigating TENS for post-stroke upper-limb motor rehabilitation, the sample size was derived using a moderate standardized effect size (*d* = 0.5) on the primary endpoint (FMA-UE), two-sided α = 0.05, and power (1–β) = 0.80. The required sample is 128 participants (32 per group). Allowing for ~20% attrition, the target enrollment is 156 (39 per group).

A total of 156 participants will be recruited from the Rehabilitation Department of Shenzhen Bao'an District Fuyong People's Hospital. Inclusion criteria are: (1) age 40–80 years; (2) clinically confirmed ischemic or hemorrhagic stroke 10 days to 2 months prior to enrollment; (3) Brunnstrom stage III–V for upper-limb motor recovery (mild to moderate impairment); (4) baseline FMA-UE 15–55; (5) able to perform ≥ Grade 2 voluntary movement (gravity-eliminated) in the paretic upper limb on MMT.

Exclusion criteria include: (1) any uncontrolled or unstable medical, cardiovascular, neurological, or orthopedic condition that could interfere with rehabilitation or study participation; (2) contraindications to TENS (e.g., implanted pacemaker, metal near the stimulation site, active skin lesions, known electrode allergy); (3) severe receptive aphasia, MoCA < 20 indicating moderate–severe cognitive impairment, or any psychiatric illness that could interfere with understanding instructions or providing informed consent; (4) clinically significant peripheral neuropathy of the paretic upper limb, diagnosed clinically or via electrophysiology; (5) concurrent participation in another interventional clinical trial or use of investigational drugs; (6) severe contractures/deformities or fixed joint limitations of the shoulder, elbow, wrist, or fingers that preclude voluntary movement or electrode placement; (7) Brunnstrom stage I–II of the upper limb.

### Recruitment strategies

Participant recruitment for this study will be conducted through coordinated efforts within our hospital, affiliated departments, and an extensive network of community healthcare centers. The Rehabilitation Department, which houses 54 inpatient beds, maintains a consistently high occupancy rate, with approximately 75% of admitted patients presenting with post-stroke upper limb motor dysfunction—providing a robust pool of eligible candidates. Physicians, nurses, and physical therapists will proactively identify and approach potential participants during routine clinical care and rehabilitation sessions.

To further expand recruitment, we will leverage our established collaborations with the Neurology and Neurosurgery Departments, ensuring a continuous referral stream of stroke patients. In addition, our hospital operates 30 community healthcare centers, collectively serving a population of over 1 million residents. These centers play a pivotal role in early post-stroke management and follow-up, offering a substantial source of eligible participants from the subacute and chronic phases of recovery. Recruitment through these centers will involve physician-led patient education, targeted referral protocols, and coordinated outreach between community physicians and the research team.

Supplementary strategies will include informational posters placed in high-traffic areas of both the hospital and community centers, distribution of study leaflets during routine health education sessions, and public awareness initiatives aimed at stroke patient support groups. These multi-level, cross-sector approaches are designed to maximize study visibility, ensure adequate enrollment, and maintain diversity in participant demographics to enhance the generalizability of the findings.

### Enrollment procedures

Participants will be enrolled in batches of approximately 20–30 to facilitate scheduling and to maintain balanced allocation across study arms via stratified block randomization. During the 3 weeks prior to randomization, baseline assessments will be completed for each batch to confirm eligibility.

Before any study-specific procedures, the Principal Investigator (PI) or a designated study coordinator will obtain written informed consent after explaining the trial's objectives, procedures, and potential risks and benefits. Eligibility screening, based on predefined inclusion and exclusion criteria, will be conducted by trained personnel. To minimize attrition and support adherence, participants will receive advance scheduling information, regular reminders, and logistical support as needed.

### Randomization

After written informed consent and baseline assessments, participants will be randomized by an off-site research assistant using a computer-generated sequence in SPSS (fixed permuted blocks of 8) in a 1:1:1:1 ratio to 2 Hz TENS, 100 Hz TENS, placebo TENS, or a no-TENS usual-care control. Allocation concealment will be ensured with sequentially numbered, opaque, sealed envelopes prepared by personnel independent of enrollment and assessment. Stratified block randomization will be used by Brunnstrom stage (III/IV/V), sex (male/female), stroke type (ischemic/hemorrhagic), and lesion side (left/right). Outcome assessors will remain blinded to group assignment.

To support participant blinding between active and sham arms, the placebo TENS device will be identical in appearance and procedures, will deliver a brief ramp-up to a just-perceptible tingling (~20–30 s), and will then fade to an imperceptible output while maintaining visual cues. Participants in the no-TENS control arm will not be blinded; TENS operators cannot be blinded due to device setup and will not perform outcome assessments. Electrode placement will follow a predefined protocol by Brunnstrom stage (e.g., wrist extensors in stage III; finger extensors/supinators in stage IV; intrinsic hand muscles in stage V). All operators will receive standardized training to ensure consistent application.

### Statistical analysis

All analyses will be performed in SPSS 27.0 on the intention-to-treat set. Baseline characteristics will be compared with one-way ANOVA or Kruskal–Wallis tests (continuous) and chi-square or Fisher's exact tests (categorical). The primary endpoint is FMA-UE; the primary comparison is 100 Hz vs. 2 Hz at week 8. Longitudinal changes will be analyzed using a linear mixed-effects model with random intercepts (participants), fixed factors group (2 Hz, 100 Hz, placebo, control) and time (baseline, week 4, week 8, 1 month post-discharge, 3 months post-discharge), and the group × time interaction; prespecified covariates (age, sex, time since stroke, stroke type, side of hemiplegia) will be included. Other pairwise contrasts will be adjusted using the Holm method; Brunnstrom-stage subgroup analyses will be exploratory via interaction terms. Secondary continuous outcomes will use the same LMM; ordinal outcomes (MMT, MAS, Lindmark) will use proportional-odds mixed models. Missing data will not use LOCF; inference will rely on the LMM under MAR with multiple-imputation sensitivity.

### Study intervention

The three intervention arms-−2 Hz TENS, 100 Hz TENS, and placebo TENS—will each receive 24 treatment sessions over 8 weeks (3 sessions/week, 30 minutes/session). This dosing schedule is informed by prior randomized trials in stroke rehabilitation (24). To support participant blinding between active and sham, the placebo TENS device will be identical in appearance and procedures, will deliver a brief ramp-up to a just-perceptible tingling (~20–30 s), and will then fade to an imperceptible output while maintaining visual cues. no therapeutic current will be delivered thereafter. The no-TENS usual-care control arm will receive standardized conventional rehabilitation therapy only (no TENS or other neuromodulation) during the study period.

### Conventional rehabilitation therapy

All participants will receive standardized conventional rehabilitation therapy throughout the intervention period, in line with routine clinical practice. This will include physical therapy (PT) focused on upper-limb motor training and basic activities of daily living (ADL) practice. Upper-limb PT sessions will emphasize active-assisted range of motion, strengthening exercises, and task-specific motor re-education based on each patient's functional level. ADL training will involve simulated daily tasks such as grooming, dressing, and reaching.

Therapy will be delivered 5 days per week, with each session lasting approximately 40 minutes. The content and intensity of conventional rehabilitation will be standardized across all study groups to reduce potential confounding effects. All PT sessions will be provided by licensed therapists following hospital rehabilitation protocols. Attendance and adherence will be documented daily using rehabilitation checklists, and weekly compliance summaries will be reviewed by study coordinators. No other forms of neuromodulation (e.g., neuromuscular electrical stimulation [NMES], repetitive transcranial magnetic stimulation [rTMS], or electroacupuncture) will be permitted during the trial period to isolate the effect of TENS; otherwise, all groups will receive the same standardized PT/ADL program. Any protocol deviations, including non-adherence to conventional therapy, will be tracked and reported in the final CONSORT flow diagram in accordance with CONSORT 2010 guidelines.

### Intervention details

TENS will be delivered using the KD-2A (Beijing Yaoyang Kangda Medical Instruments Co., Ltd.). Parameters will be standardized across active groups: pulse duration 300 μs (0.3 ms), frequency 2 Hz or 100 Hz, and 30-min sessions. Current intensity will be individually titrated to produce a strong but comfortable paresthesia below the motor threshold (no visible contraction) for both frequencies. Intensity will be ramped over ~5–10 s at the start and re-checked every ~5 min to maintain a consistent perceived intensity (e.g., NRS 5–7), with adjustments limited by comfort and device specifications. Session-level intensity (mA) and any adjustments will be recorded for fidelity monitoring.

**Figure 2:**
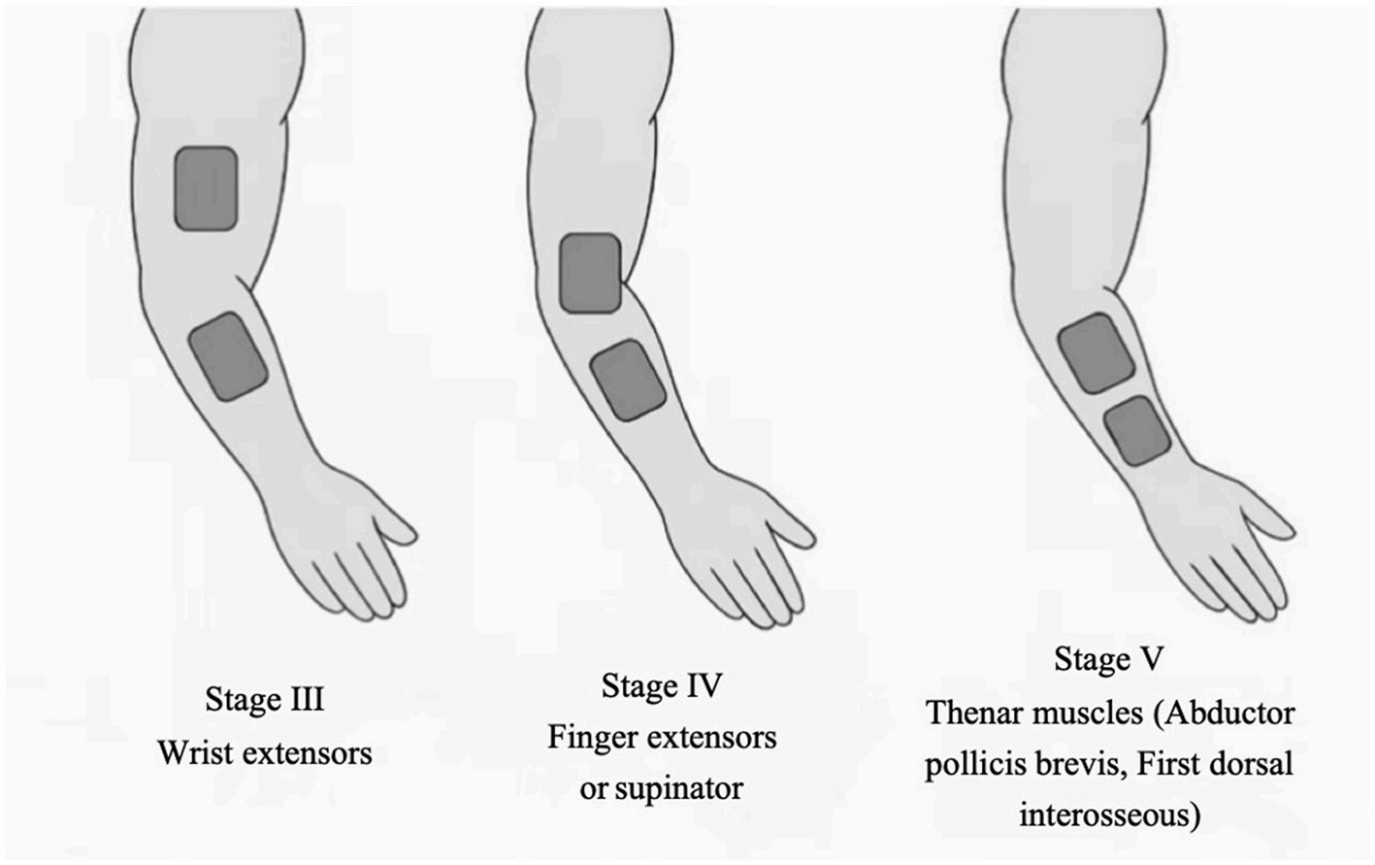
Electrode placement strategies for upper limb muscles based on Brunnstrom stages III–V.

Electrode placement will be individualized based on each participant's Brunnstrom stage of upper-limb recovery, targeting muscle groups most in need of facilitation at that stage on the paretic limb. In stage III, where synergistic movement patterns dominate and voluntary control begins to emerge, electrodes will be placed over the wrist extensors, which are typically weak and essential for functional hand use (25). In stage IV, as spasticity declines and movement outside synergy becomes possible, stimulation will focus on finger extensors or forearm supinators to promote distal control (26). In stage V, with increasing isolated joint movements, intrinsic hand muscles (e.g., thenar muscles) will be targeted to enhance fine motor skills and dexterity (27). Unless otherwise indicated, pairs of self-adhesive surface electrodes will be positioned longitudinally over the muscle belly with an inter-electrode distance of approximately 2–3 cm, guided by anatomical landmarks. Recommended placements and procedural guidance for each Brunnstrom stage are illustrated in Figure 2 and detailed in Table 2.

**Table 2:** Electrode placement strategy by Brunnstrom Stage.

**Brunnstrom stage**	**Stage III**	**Stage IV**	**Stage V**
Target muscle(s)	Wrist extensors (Extensor carpi radialis, Extensor carpi ulnaris)	Finger extensors (Extensor digitorum), Supinator	Thenar muscles (Abductor pollicis brevis), First dorsal interosseous
Origin & Insertion	Lateral supracondylar ridge of humerus → 2nd & 5th metacarpals	Lateral epicondyle of humerus → Extensor expansion; Lateral epicondyle → Radius	Flexor retinaculum/scaphoid/trapezium → Proximal phalanx of thumb; 1st & 2nd metacarpals → Proximal phalanges
Innervation	Radial nerve (C6–C8)	Radial nerve (C6–C8)	Median nerve (C8–T1); Ulnar nerve (C8–T1)
Electrode placement	Over muscle belly of wrist extensors, ~2–3 cm apart on dorsal forearm	On dorsal forearm for finger extensors; lateral forearm for supinator	Thenar eminence and radial side of palm for intrinsic hand muscles
Clinical notes	Facilitates breaking flexor synergy, improves voluntary wrist extension	Promotes distal activation, supports functional reach and release	Enhances fine motor control, crucial for object manipulation tasks

Placebo TENS will use an identical device with the indicator light on and electrodes in the same positions as the active arms. To support participant blinding between active and sham, the sham unit will deliver a brief ramp-up to a just-perceptible tingling (~20–30 s) and then will fade to an imperceptible output while maintaining the visual cues; no therapeutic current will be delivered thereafter.

### Procedure

All outcome assessments will be conducted by an experienced rehabilitation therapist at five time points: baseline (prior to the intervention), mid-intervention (week 4), post-intervention (week 8), and at 1-month and 3-month follow-up visits.

## Primary outcome

### Fugl-Meyer assessment of upper extremity

The FMA-UE is a widely used and highly reliable tool for evaluating upper limb motor control in individuals who have experienced a stroke (27). It provides a comprehensive assessment of motor recovery, focusing on proximal-to-distal movements and the transition from synergistic to isolated movement patterns. The FMA-UE is divided into four subsections: shoulder-arm, wrist, hand, and coordination and speed. The assessment consists of 33 items, with each item scored on an ordinal scale from 0 to 2, where 0 indicates no performance and 2 indicates full performance, resulting in a maximum total score of 66 (28).

The FMA-UE has demonstrated excellent intrarater reliability (ICC = 0.997) and interrater reliability (ICC = 0.993) (29), making it a highly reliable tool for assessing motor function in stroke patients. This assessment is particularly valuable for tracking recovery over time, as it allows clinicians to quantify the progression of motor function from the shoulder to the hand, enabling detailed characterization of motor recovery patterns across multiple joint segments, which is critical for evaluating rehabilitation outcomes in stroke patients. It has been validated in numerous studies and is widely accepted in clinical research for evaluating the effectiveness of rehabilitation interventions.

### Secondary outcomes

(1) Muscle strength of wrist flexion and extension on the paretic upper limb will be assessed using MMT on a 0–5 ordinal scale (0 = no contraction; 5 = normal strength). When performed by trained clinicians, MMT has shown good inter-rater reliability and is widely used to quantify segmental muscle weakness in both clinical and research settings (30).

(2) Spasticity of the wrist flexors on the paretic upper-limb will be assessed using the MAS, which rates resistance to passive movement on a 6-point ordinal scale (0, 1, 1+, 2, 3, 4; higher scores indicate greater hypertonia). Assessments will follow standardized positioning and stretch velocity. The MAS has demonstrated acceptable intra-rater reliability in upper-limb hemiparesis and remains a widely used tool for monitoring changes in muscle tone during stroke rehabilitation (31).

(3) Grasp function of the paretic hand will be assessed using the hand subscale of the Lindmark Motor Score. Participants will be asked to grasp and hold a tennis ball; performance will be rated on a 4-point ordinal scale: 0 = unable to grasp; 1 = grasp achieved but cannot resist slight pressure; 2 = maintains grip for ≥5 s with mild incoordination under moderate resistance; 3 = grasps, holds, and releases smoothly under strong resistance. This test captures hand strength and coordination and will contribute to the evaluation of distal fine-motor function after stroke (32).

(4) Functional independence in ADLs will be assessed using the Barthel Index, which covers 10 basic ADL domains with a total score of 0–100 (higher scores indicate greater independence). The Barthel Index has demonstrated excellent inter-rater reliability and extensive validation in stroke populations and will serve as a robust indicator of post-stroke functional recovery and self-care capacity (33).

(5) Cortical excitability and interhemispheric balance will be evaluated with resting-state EEG. Signals will be recorded according to the international 10–20 system, focusing on C3 and C4 over the sensorimotor cortices, and processed with standard preprocessing (artifact rejection and filtering). Frequency-domain analyses will extract α (8–13 Hz) and β (13–30 Hz) band power; the C3/C4 α/β power ratio and interhemispheric asymmetry indices will be computed. These EEG metrics have been widely used and validated as non-invasive markers of motor-cortex activation, hemispheric balance, and neuroplastic change, and will provide complementary mechanistic insight into the effects of TENS on motor-system reorganization (34).

### Adherence

To enhance adherence and optimize rehabilitation outcomes in participants with post-stroke upper-limb motor dysfunction, a structured in-hospital management protocol will be adopted. First, a ward-based scheduling policy will be implemented, requiring participants to remain under inpatient rehabilitation care throughout the intervention period, with medically necessary exceptions, thereby minimizing interruptions and ensuring timely clinical supervision. Second, an attendance-tracking system will be implemented using individualized rehabilitation treatment cards, which will be signed by therapists upon completion of each session to ensure consistent participation. Third, nurses will play a central role in adherence monitoring and education, providing bedside instructions, printed materials, and video-based demonstrations, and conducting daily checks to identify and address potential barriers to engagement. Fourth, a multidisciplinary rehabilitation team, including physicians, therapists, and nurses, will provide personalized interventions tailored to participants' functional status and Brunnstrom stage. Weekly case conferences will be held to review adherence, monitor progress, and adjust treatment strategies accordingly. Finally, psychological support measures, including motivational interviewing and involvement of family members, will be integrated to reinforce treatment motivation, reduce resistance, and promote sustained engagement. Collectively, this multifaceted strategy is intended to strengthen participant adherence and support functional outcomes.

### Follow-up timepoints and assessment procedures

Follow-up assessments are scheduled at 1 month and 3 months after completion of the 8-week intervention. The selection of these timepoints was based on both clinical and neurophysiological considerations (35). The 1-month follow-up corresponds to the early consolidation phase of motor recovery, during which short-term neuroplastic changes may stabilize or continue to evolve. The 3-month timepoint is commonly used in stroke rehabilitation research to evaluate the sustainability of treatment effects, as it reflects a period when spontaneous recovery tends to plateau and long-term functional gains—if present—can be more clearly attributed to the intervention. These intervals are supported by previous randomized controlled trials examining post-stroke motor rehabilitation outcomes.

All follow-up evaluations will be conducted in person at the Rehabilitation Department of Shenzhen Bao'an District Fuyong People's Hospital. Assessments will be performed by certified physical and occupational therapists with at least 3 years of clinical experience in stroke rehabilitation. To ensure consistency and reliability across timepoints, all assessors will undergo structured training in the administration and scoring of each outcome measure prior to participant enrollment. Inter-rater reliability will be reinforced through pilot calibration sessions and ongoing periodic reviews. The same assessor will be assigned to each participant across baseline and follow-up assessments whenever feasible, to minimize inter-observer variability. All assessment procedures will follow a standardized operating protocol and will be documented using unified case report forms.

### Safety

Participants will be monitored weekly throughout the 8-week intervention and at the 1- and 3-month follow-up visits for any adverse events (AEs), defined as any undesirable medical occurrence during the study, regardless of its relationship to the intervention. All AEs will be recorded on a standardized case report form (CRF), graded for severity (mild, moderate, severe), and assessed for relatedness to study procedures (unrelated, unlikely, possible, probable, definite). Events will be categorized and periodically analyzed for trends that might indicate safety concerns; particular attention will be given to serious adverse events (SAEs) or events deemed at least possibly related to the TENS intervention. SAEs will be reported to the Principal Investigator and the ethics committee within 24 h, in accordance with institutional policy and the DSMB charter. Safety oversight will be conducted under the protocol approved by the institutional ethics committee.

### Data management

Study data will be recorded on paper/electronic case report forms (CRFs). Double data entry or independent verification will be used; built-in range/logic checks will flag discrepancies for resolution against source documents. Personally identifying information will be stored separately from analysis datasets; analytical files will contain de-identified records referenced only by unique study IDs. Role-based permissions, audit trails, and time-stamped change logs will be maintained. Physical CRFs will be stored in locked cabinets in the PI's or study coordinator's office; electronic data will be encrypted and backed up regularly. After database lock, data will be exported to SPSS 27.0 for statistical analysis.

### Data and safety monitoring

An independent DSMB will be established and approved prior to trial initiation by the Department of Rehabilitation Medicine at Fuyong People's Hospital, Bao'an District, Shenzhen. The DSMB will comprise experts in post-stroke motor rehabilitation, clinical trial methodology, statistical design, and adverse-event monitoring. All members will be unaffiliated with the study to ensure impartial oversight.

The DSMB will be responsible for:

1. Monitoring study progress to ensure adherence to the approved protocol;
2. Evaluating participant safety through ongoing review of adverse events and safety concerns;
3. Assessing data quality, completeness, and integrity;
4. Recommending continuation, modification, or termination of the trial based on interim analyses.

The Board will convene at least once during the study, with additional meetings held as needed. All decisions will be made by majority vote. After each meeting, the DSMB Chair will submit a formal report to the Principal Investigator (PI), who will forward it to the Director of the Department of Rehabilitation Medicine. All DSMB discussions and materials will be treated as strictly confidential. The DSMB Chair will receive reports of all serious adverse events (SAEs), including notifications within 24 h of any SAE or death, annual summaries of adverse events, and periodic progress reports prior to each meeting.

### Analysis

The primary outcome will be the change in FMA–UE from baseline to week 4, week 8, 1 month post-discharge, and 3 months post-discharge. The primary contrast will be 100 Hz vs. 2 Hz at week 8; other time-point contrasts will be supportive/exploratory.

A prespecified subgroup analysis by Brunnstrom stage (III/IV/V) will examine whether frequency effects differ by motor-recovery stage (exploratory interpretation). Within the FMA–UE, we will also explore segment/domain effects (proximal vs, distal components and the coordination/speed subscale).

Secondary outcomes will include MMT (wrist flexor/extensor strength), MAS (wrist-flexor spasticity), Lindmark Motor Score—hand subscale (grasp), and the Barthel Index (ADLs). These measures complement the FMA–UE by capturing muscle strength, spasticity, hand function, and functional independence.

## Discussion

This protocol describes a randomized controlled trial comparing low-frequency (2 Hz) and high-frequency (100 Hz) TENS for post-stroke upper-limb motor recovery. To our knowledge, it is among the earliest head-to-head evaluations of TENS frequency and the first in our setting to use a four-arm design that includes both a placebo TENS arm and a no-TENS usual-care control. Although TENS is used in stroke rehabilitation, current guidance does not establish frequency-specific parameters for upper-limb recovery, and existing findings are mixed. By directly contrasting two commonly used frequencies under controlled conditions, this study aims to generate frequency-dependent evidence to inform clinical practice and support more individualized neuromodulation strategies.

TENS is widely used in neurorehabilitation, yet its mechanisms of action vary considerably with stimulation frequency (36). Low-frequency TENS (e.g., 2 Hz) has been associated with activation of endogenous opioid pathways and may modulate spasticity or pain through long-term depression (LTD)-like mechanisms (37). In contrast, high-frequency TENS (e.g., 100 Hz) appears to engage non-opioid descending inhibitory systems and enhance corticospinal excitability, aligning more closely with long-term potentiation (LTP)-like plasticity (38). Previous studies have yielded inconsistent results: some report minimal or short-term effects with low-frequency TENS, while others show sustained motor improvements with higher frequencies. However, these studies often differ in design, sample characteristics, or lack adequate controls, limiting the generalizability of their findings.

A key strength of this study lies in its individualized stimulation strategy guided by the Brunnstrom stages of motor recovery. Electrode placement will be tailored to target muscle groups that are most functionally relevant at each stage—for example, stimulating wrist extensors in stage III to disrupt flexor synergy, finger extensors and supinators in stage IV to promote distal coordination, and intrinsic hand muscles in stage V to enhance fine motor control. This stage-specific approach reflects the neurorehabilitation principle that task-relevant, peripheral sensory input can facilitate use-dependent plasticity and promote reorganization of motor pathways. By aligning stimulation targets with the evolving motor status of the patient, the protocol aims to optimize the neuromodulatory effects of TENS in a phase-specific and personalized manner.

The inclusion of both a placebo group and a no-TENS usual-care control group strengthens the internal validity of this trial by enabling a clearer distinction between true neuromodulatory effects and nonspecific influences such as attention, expectation, or spontaneous recovery. Moreover, stratified randomization based on Brunnstrom stage, sex, stroke type, and lesion side was employed to ensure balanced baseline characteristics across groups, further reducing the risk of allocation bias and enhancing the reliability of outcome comparisons.

Despite its methodological strengths, this study has several limitations. First, as a single-center trial, the generalizability of the findings may be limited due to potential center-specific clinical practices or population characteristics. Second, neurophysiological measures such as transcranial magnetic stimulation (TMS), or functional magnetic resonance imaging (fMRI) were not included, restricting our ability to explore the underlying cortical mechanisms of TENS-induced motor recovery. Third, although the follow-up period extends to 3 months post-intervention, this duration may be insufficient to evaluate long-term outcomes or the sustainability of treatment effects. Lastly, while the sample size is adequately powered for primary comparisons, it may be insufficient for detecting significant differences in subgroup analyses, particularly those stratified by Brunnstrom stage.

Future research should incorporate neurophysiological biomarkers—such as TMS, or fMRI—to directly examine the cortical mechanisms underlying different TENS frequencies. In addition, longer-term follow-up and multicenter trials are warranted to validate the present findings and improve their generalizability across diverse clinical settings. Exploring the synergistic potential of TENS in combination with other neuromodulatory interventions, such as transcranial direct current stimulation (tDCS) or robotic-assisted therapy, may also offer promising avenues for enhancing post-stroke motor recovery.

In conclusion, this trial addresses an important gap in post-stroke rehabilitation by systematically comparing the effects of low- and high-frequency TENS on upper limb motor recovery using a rigorously controlled design. Leveraging a broad recruitment network spanning both hospital and community settings, and incorporating stratified analyses based on Brunnstrom stages, the findings are expected to generate frequency-specific evidence with strong clinical applicability. These results may inform future clinical guidelines, support individualized stimulation protocols, and contribute to integrated rehabilitation strategies that not only improve motor performance but also enhance functional independence and quality of life in stroke survivors.
